# Ecological weed management and square planting influenced the weed management, and crop productivity in direct-seeded rice

**DOI:** 10.1038/s41598-024-56945-y

**Published:** 2024-05-06

**Authors:** Mona Nagargade, Manoj Kumar Singh, Vishal Tyagi, Prabhu Govindasamy, Anil K. Choudhary, Kuldeep Rajpoot, Adarsh Kumar, Preeti Singh, Debalin Sarangi

**Affiliations:** 1 Agronomy, ICAR-Indian Agricultural Research Institute, New Delhi, 110012 India; 2 Department of Agronomy, Institute of Agricultural Sciences, Banaras Hindu University, Uttar Pradesh, Varanasi, 221005 India; 3 ICAR-National Research Centre for Banana, Tiruchirappalli, 620 102 India; 4 ICAR-Central Potato Research Institute, Himachal Pradesh, Shimla, 171001 India; 5 ICAR-National Bureau of Agriculturally Important Microorganisms, Mau, Uttar Pradesh 275101 India; 6 ICAR- Indian Agricultural Research Institute, Jharkhand, 825405 India; 7 University of Minnesota, Minneapolis, MN 55108-6026 USA

**Keywords:** Ecology, Plant sciences

## Abstract

Herbicide use may pose a risk of environmental pollution or evolution of resistant weeds. As a result, an experiment was carried out to assess the influence of different non-chemical weed management tactics (one hoeing (HH) at 12 DAS followed by (*fb*) one hand weeding at 30 DAS, one HH at 12 DAS *fb* Sesbania co-culture and its mulching, one HH at 12 DAS *fb* rice straw mulching @ 4t ha^−1^, one HH at 12 DAS *fb* rice straw mulching @ 6 t ha^−1^) on weed control, crop growth and yield, and economic returns in direct-seeded rice (DSR). Experiment was conducted during kharif season in a split-plot design and replicated thrice. Zero-till seed drill-sown crop (PN) had the lowest weed density at 25 days after sowing (DAS), while square planting geometry (PS) had the lowest weed density at 60 DAS. PS also resulted in a lower weed management index (WMI), agronomic management index (AMI), and integrated weed management index (IWMI), as well as higher growth attributes, grain yield (4.19 t ha^–1^), and net return (620.98 US$ ha^–1^). The cultivar Arize 6444 significantly reduced weed density and recorded higher growth attributes, yield, and economic return. In the case of weed management treatments, one HH at 12 DAS *fb* *Sesbania* co-culture and its mulching had the lowest weed density, Shannon-weinner index and eveness at 25 DAS. However, one hoeing at 12 DAS *fb* one hand weeding at 30 DAS (HH + WH) achieved the highest grain yield (4.85 t ha^–1^) and net returns (851.03 US$ ha^–1^) as well as the lowest weed density at 60 DAS. PS × HH + WH treatment combination had the lowest weed persistent index (WPI), WMI, AMI, and IWMI, and the highest growth attributes, production efficiency, and economic return.

## Introduction

On a global scale, rice is one of the stable food crops cultivated on around 165.25 million hectares, with a production of 787.29 million tonnes^1^. India is a major producer and consumer of rice, accounting for 27.27% of the global rice cultivated area (45.07 million hectares) and 15.79% of production^2^. Global rice demand is expected to rise by more than 40% by 2050 to fulfil the needs of the world's growing population^3^. Transplanted rice is still the most common and traditional planting method in India, requiring a large amount of natural resources and nonrenewable energy sources. As a consequence, meeting the ever-increasing demand for rice in the context of dwindling natural resources is a big concern^4^. Direct Seeded Rice (DSR) is an alternative choice for rice growers around the globe in the face of limited water and energy resources^5^. However, weed infestation is a major hurdle to the successful deployment of DSR. Weed competition throughout the season results in 100% yield reduction in DSR^6^. In general, the critical weed-free period for DSR goes from 11 to 83 DAS, which is longer than for transplanted rice^7^. Effective weed management in direct-seeded rice necessitates a multifaceted strategy. Many authors suggested that it is challenging to effectively manage weeds in DSR with a single strategy^8,9^. In this context, adopting the Integrated Weed Management (IWM) strategy, which involves the synergistic integration of at least two components, emerges as a successful approach capable of addressing this challenge comprehensively^10^. A more competitive crop has an edge over weeds and lowers weed-related yield losses^11–13^.

Adoption of short-duration, highly competitive, and high-yielding rice hybrids would be more cost-effective in northern India's productive Gangetic plains, where the rice–wheat cropping system (RWCS) persists. The early vigor and phenotypic flexibility of short-duration high-yielding rice hybrids demand the right geometry for a better DSR establishment. It is widely acknowledged that wider spacing and single rice transplanting in the system of rice intensification (SRI) result in profuse tillering and higher yield^14^. Wider spacing in hybrids reduces seed rate and production cost^12,15^. The wider spacing also facilitates mechanical weed control in the spaces between rows; however, it takes a long time for the canopy to close compared to the narrower rows^16,17^, this leads to a longer crucial period for weed management. However, weed-competitive hybrids quickly close the canopy, provide shade, and prevent weed growth^18^. Hybrids are more vigorous than inbred; therefore, the weed suppression ability of hybrids may be utilized in DSR rice. In general, the recommended seed rate for inbred varieties shown in zero-till seeders or dry direct-seeded method is 25–30 kg ha^–1^ in the DSR system^5^. The seed rate, like inbred lines, increases production costs and reduces the net return in hybrids. Therefore, DSR uses square planting geometry to provide identical growing conditions and better weed management like SRI in rice. There are opportunities to use cultural practices for better weed management in DSR^19^. There is a clear association between weed emergence time and crop yield loss^13^. Yield losses are greater when weeds emerge earlier or simultaneously with the crop^20^. Despite chemical weed control methods achieved distinctive successes for weed management in field crops, herbicide use could pose hazards in the environment. Therefore, mechanical and manual weed control methods are still preferred. A hoe at crop emergence may suppress weeds that germinate early, providing a competitive edge to rice seedlings. Manual weeding is the most common technique in rice; however, it is tedious and not economically feasible^21^. The combination of hoe and hand weeding is most appropriate, especially for small farms and places where laborers are cheap^22^. Also, the integration between mechanical and cultural or chemical methods exhibited better weed control efficiency than the use of individual practice^23^.

The soil organic carbon loss is a major concern in tropical regions due to rapid mineralization^24^. The inclusion of fast-growing nitrogen-fixing crops as cover crops or co-cultures might enhance the organic carbon content of the soil and improve the available nutrient status^25^. Co-cultivation of fast-growing crops like *Sesabnia aculeata* will suppress the weeds and add nitro-gen to the soil. According to Gill and Walia^26^, the use of *S. aculeate* residues conserves soil moisture and adds roughly 15 kg N ha^–1^. The *S. aculeata* intercropping greatly reduce the weed density and biomass in DSR due to the low light transmission^27^. Residue retention enhances soil health by restoring the soil's physical characteristics^28^, and enhancing microbial activity and nutrient cycling^29^.

Mulching crop residue is a promising practice to suppress weed emergence and conserve moisture; however, this is almost impossible for large farms. The amount of rice straw is plentiful in the RWCS of northern India, and generally farmers burn the residues for easier and faster wheat planting^30^. The burning of residues pollutes the environment by emitting gases that are hazardous to human and environmental health^5^. There are several advantages in retaining rice residue. Rice straw can last longer in the field due to its higher lignin and silica content, which can help in managing weeds^31^. Furthermore, rice straw contains nutrients that can be recycled and utilized as organic fertilizer^32,33^, to improve soil fertility^34,35^. Retaining rice residue mulch provide an environmentally sound solution for managing weeds^13,24^. However, research on the effect of rice cultivars, planting geometry, and non-chemical weed management on weed density, diversity, and performance of hybrid rice cultivars is sparse.

Knowledge in these areas would make ecological weed management easier. To accomplish this, we hypothesise that the integration of cultivars, crop geometry, and non-chemical weed management approaches will result in sustainable rice production and weed management as well as healthy environment. The outcomes of this research can significantly contribute to the advancement of sustainable and economically viable agricultural practices. Therefore, a study was conducted with the following objectives: (1) to assess the influence of planting geometry, cultivars, and non-chemical weed management on crop performance, weed density and diversity of hybrid rice production (2) to analyze the economic implications of implementing these approaches in hybrid rice production. These objectives aim to offer valuable insights that can guide the development of economically viable and sustainable weed management practices in DSR.

## Results and discussion

### Weed flora

During the two-year study period, fourteen weed species from six different families were recorded at the experimental site. Among these, six species were grasses [i.e., bermudagrass (*Cynodon dactylon* (L.) Pers*.*), large crabgrass (*Digitaria sanguinalis* (L.) Scop)*,* jungle rice (*Echinochloa colona* (L.) Link), barnyardgrass (*Echinochloa crus-galli* (L.) P. Beauv), goosegrass (*Eleusine indica* (L.) Gaertn.)*,* and torpedograss (*Panicum repens* L.), five species were broadleaves [ i.e., blistering ammannia (*Ammannia baccifera* L.), pink node flower (*Caesulia axillaris* Roxb*.)*, eclipta (*Eclipta alba* (L.) Hassk.), water primrose (*Ludwigia parviflora* Roxb.), and gulf leafflower (*Phyllanthus fraternus* G.L.Webster), and three species were sedges [i.e., smallflower umbrella (*Cyperus difformis* L.), flatsedge *(Cyperus iria* L.) and grasslike fimbry (*Fimbristylis miliacea* (L.) Vahl)]. The grasslike fimbry, blistering ammannia*,* jungle rice and bermuda grass were the dominant weed species and had the highest relative densities of 12.15%, 11.66%, 11.14%, and 11.12%, respectively (Fig. S1).

### Weed density

Grasses, broadleaf, sedges, and total weed densities were significantly (p < 0.05) influenced by planting geometry, cultivars, and non-chemical weed management at 25 and 60 DAS (Table 1). At 25 DAS, grass, sedge, broadleaf, and total weed densities were 18.14%, 21.13%, 29.04% and 22.36%, lower respectively, for P_N_ compared to P_S_ geometry. In contrast, inverse occurred at 60 DAS, where P_S_ observed 23.93%, 26.64%, 18.03%, and 22.67% lower weed densities of grasses, sedges, broadleaf, and total weeds, respectively, than P_N_. Lower weed densities in P_S_ geometry may be due to weed growth smothering as a result of uniform plant-to-plant and row-to-row spacing^30,36^. Square planting further encourages crops to compete with weeds as a result of the better availability of space, light, and nutrients^18,37,38^. Nichols et al.^39^, and Dass et al.^13^, reported that a uniform row-to-row and plant-to-plant distance in rice had a lower weed-competition. Among cultivars, Arize 6444 (hybrid from Bayer) was more competitive with weeds than PHB 71 (hybrid from Pioneer) at 25 and 60 DAS. Faster emergence and robust seedlings of Arize 6444 were thought to be reasons for increased competitiveness. The cultivars that achieve early vegetative vigor and quick ground cover have a competitive advantage over weeds compared to varieties that have slow initial growth^35,40^. With regards to weed management treatments, H_H_ + S_C_ had the lowest grass, broad-leaf, sedge, and total weed densities at 25 DAS, with reductions of 90.61%, 91.81%, 89.05%, and 90.19%, respectively, compared to the weedy check.

**Table 1:** Weed density influenced by planting geometry, cultivar, and non-chemical weed management in rice.

Treatment	25 DAS				60 DAS			
	Grasses (No. m^−2^)	Sedges (No. m^−2^)	Broad-leaved weed (No. m^−2^)	Total weed density (No. m^−2^)	Grasses (No. m^−2^)	Sedges (No. m^−2^)	Broad-leaved weed (No. m^−2^)	Total weed density (No. m^−2^)
Planting geometry (PG)								
P_N_	^†^4.72 b (24.42)*	4.62 b* (23.41)	4.33 b (20.13)	8.20 b (74.51)	6.63 a (44.3)	6.50 a (42.8)	6.96 a (48.8)	12.15 a (150.0)
P_S_	5.25 a (29.83)	5.21 a (29.68)	5.14 a (28.37)	9.36 a (95.97)	5.77 b (33.7)	5.56 b (31.4)	6.33 b (40.0)	10.67 b (116.0)
* P* value	0.0027**	**0.0389**	**0.0008**	**0.0030**	**0.0140**	**0.0058**	**0.0130**	**0.0045**
Cultivar (CV)								
Arize 6444	4.73 b (24.61)	4.66 b (23.87)	4.47 b (21.68)	8.33 b (77.28)	5.99 b (36.6)	5.72 b (33.5)	6.46 b (41.9)	10.98 b (123.3)
PHB71	5.24 a (29.64)	5.18 a (29.22)	5.00 a (26.82)	9.22 a (93.20)	6.41 a (41.6)	6.33 a (40.7)	6.83 a (46.9)	11.84 a (142.8)
* P* value	**0.0064**	**0.0006**	**0.0003**	**0.0002**	**0.0054**	**0.0003**	**0.0007**	**0.0005**
Weed management (WM)								
W_C_	6.78 a (45.71)	6.57 a (42.87)	6.17 a (37.91)	11.75 a (138.19)	7.71 a (59.3)	7.62 a (58.1)	7.68 a (58.8)	13.90 a (193.9)
H_H_ + W_H_	6.10 b (36.98)	6.21 b (38.37)	5.80 b (33.43)	10.91 b (119.31)	5.24 e (27.2)	4.93 e (24.1)	5.63 e (31.3)	9.47 e (89.9)
H_H_ + S_C_	2.16 e (4.29)	1.985 e (3.51)	2.13 e (4.15)	3.70 e (13.55)	5.54 d (30.5)	5.32 d (28.1)	6.05d (36. 2)	10.20 d (104.5)
H_H_ + M_R4_	5.47 c (29.51)	5.24 c (27.19)	5.01 c (24.96)	9.41 c (88.61)	6.57 b (42.85)	6.58 b (43.07)	7.27 b (52.73)	12.37 b (153.2)
H_H_ + M_R6_	4.41 d (19.13)	4.58 d (20.79)	4.56 d (20.80)	8.12 d (66.54)	5.95 c (35.1)	5.69 c (32.3)	6.59 c (43.0)	11.10 c (123.6)
* P* value	** < 0.0001**	** < 0.0001**	** < 0.0001**	** < 0.0001**	** < 0.0001**	** < 0.0001**	** < 0.0001**	** < 0.0001**
*P* value (Interaction)								
PG × CV	0.9380	0.1439	0.5019	0.2864	0.6253	0.9096	0.4450	0.6201
PG × WM	0.4036	**0.0012**	** < 0.0001**	**0.0001**	0.2860	0.6529	** < 0.0001**	0.0651
Cv × WM	0.4134	0.7172	0.0531	0.0947	0.9310	0.7270	0.2309	0.3681
PG × CV × WM	0.8470	0.5483	**0.0282**	0.2282	0.9418	0.4785	0.0937	0.5315
Year	0.9624	0.6621	0.3227	0.5873	0.8745	0.6101	0.2172	0.6411

*The data in the parentheses are original data; means with different alphabets are significant (p < 0.05).

^†^Values shown are square-root [√(x + 0.5)] transformed means. DAS, days after sowing. P_N_, sowing with seed drill at 18.5 cm row spacing; P_S_, square planting at 25 cm × 25 cm row to row and plant to plant spacing; W_C_, weedy check (no weed management); H_H_ + W_H_, one hand hoeing at 12 DAS *fb* one hand weeding at 30 DAS; H_H_ + S_C_, one hand hoeing at 12 DAS *fb S. aculeata* co-culture and mulched 45 DAS; H_H_ + M_R4_, one hand hoeing at 12 DAS *fb* rice residue mulching @ 4 t ha^−1^; H_H_ + M_R6_, one hand hoeing at 12 DAS *fb* rice residue mulching @ 6 t ha^−1^.

**Bold P values are significant.

However, H_H_ + W_H_ treatment had the lowest weed densities at 60 DAS with 92.77%, 46.77%, 58.52% and 53.64% lower densities of grassy, broad-leaf, sedges, and total weeds compared to the weedy check. Further, treatments H_H_ + M_R4_ and H_H_ + M_R6_ recorded lower weed densities than W_C_ at both the stages. Early prevention and suppression of weed germination and growth could be the reason for the lowest weed density in the H_H_ + S_C_ treatment. Keeping the weeds free at an early stage (hand hoeing at 12 DAS) and during the peak weed emergence period (manual weeding during the active tillering stage at 30 DAS) might have resulted in reduced weed competition and weed density of all the weeds at later stages^12,18^.

**Figure 1:**
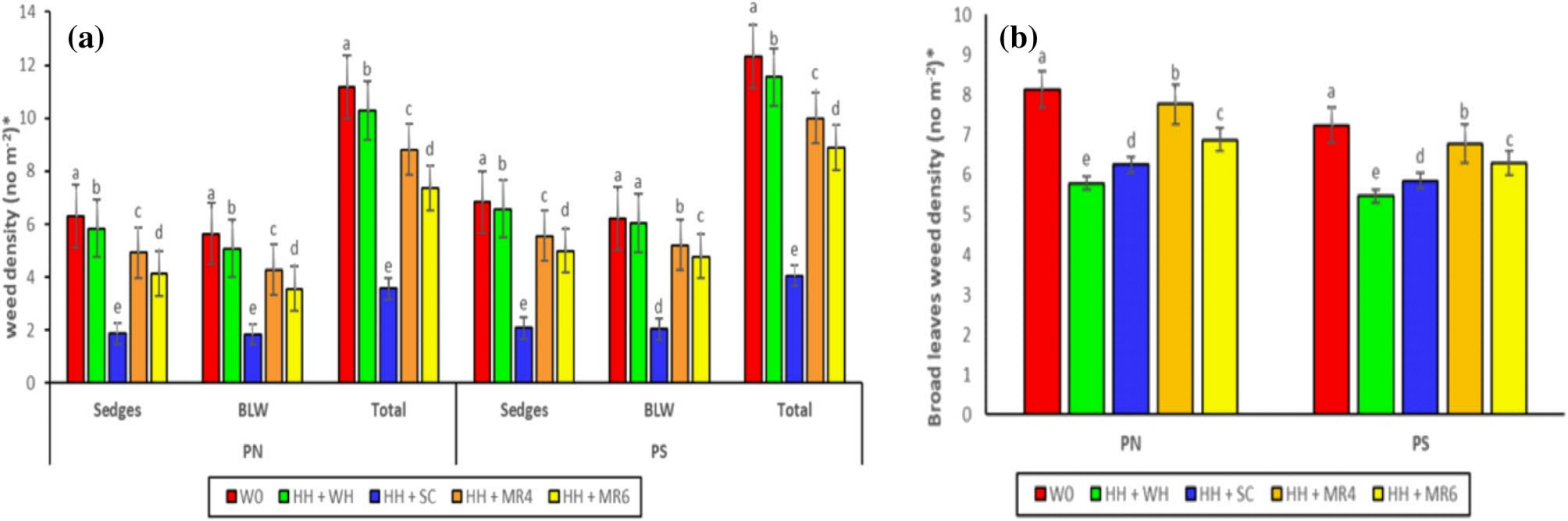
Interaction effect of planting geometry × weed management on sedge, broadleaf and total weed densities at 25 DAS (**a**) and broadleaf density at 60 DAS (**b**). PN, sowing with seed drill at 18.5 cm row spacing; PS, square planting at 25 cm × 25 cm row to row and plant to plant spacing; W0, weedy check (no weed management); HH + WH, one hand hoeing at 12 DAS fb one hand weeding at 30 DAS; HH + SC, one hand hoeing at 12 DAS fb *Sesbania aculeata* co-culture and mulched 45 DAS; HH + MR4, one hand hoeing at 12 DAS fb rice residue mulching @ 4 t ha^−1^; HH + MR6, one hand hoeing at 12 DAS fb rice residue mulching @ 6 t ha^−1^. Means with different alphabets are significant (P < 0.05). Values shown in the figure are square-root [√(x + 0.5)]-transformed means.

Interaction of planting geometry (PG) × cultivar (CV), CV × weed management (WM), and PG × CV × WM did not influence the weed density at 25 and 60 DAS (Table 1). However, planting geometry × weed management significantly (p < 0.05) influenced the broadleaf, sedge, and total weed densities at 25 DAS, and broadleaf weed density at 60 DAS (Table 1). The interaction of PG × WM revealed that P_N_ and H_H_ + S_C_ combinations resulted in the lowest broadleaf, sedge, and total weed densities (Fig. 1a,b). This could be due to the lack of space available for weed growth in close spacing and smothering effect of *sesbania* co-culture treatments^18,41^. The lowest broadleaf density at later stage under P_S_ and H_H_ + W_H_ combinations may be due to keeping the plots weed free in hand hoeing *fb* hand weeding treatments and faster crop growth when planted in the square pattern.

### Weed diversity indices

**Figure 2:**
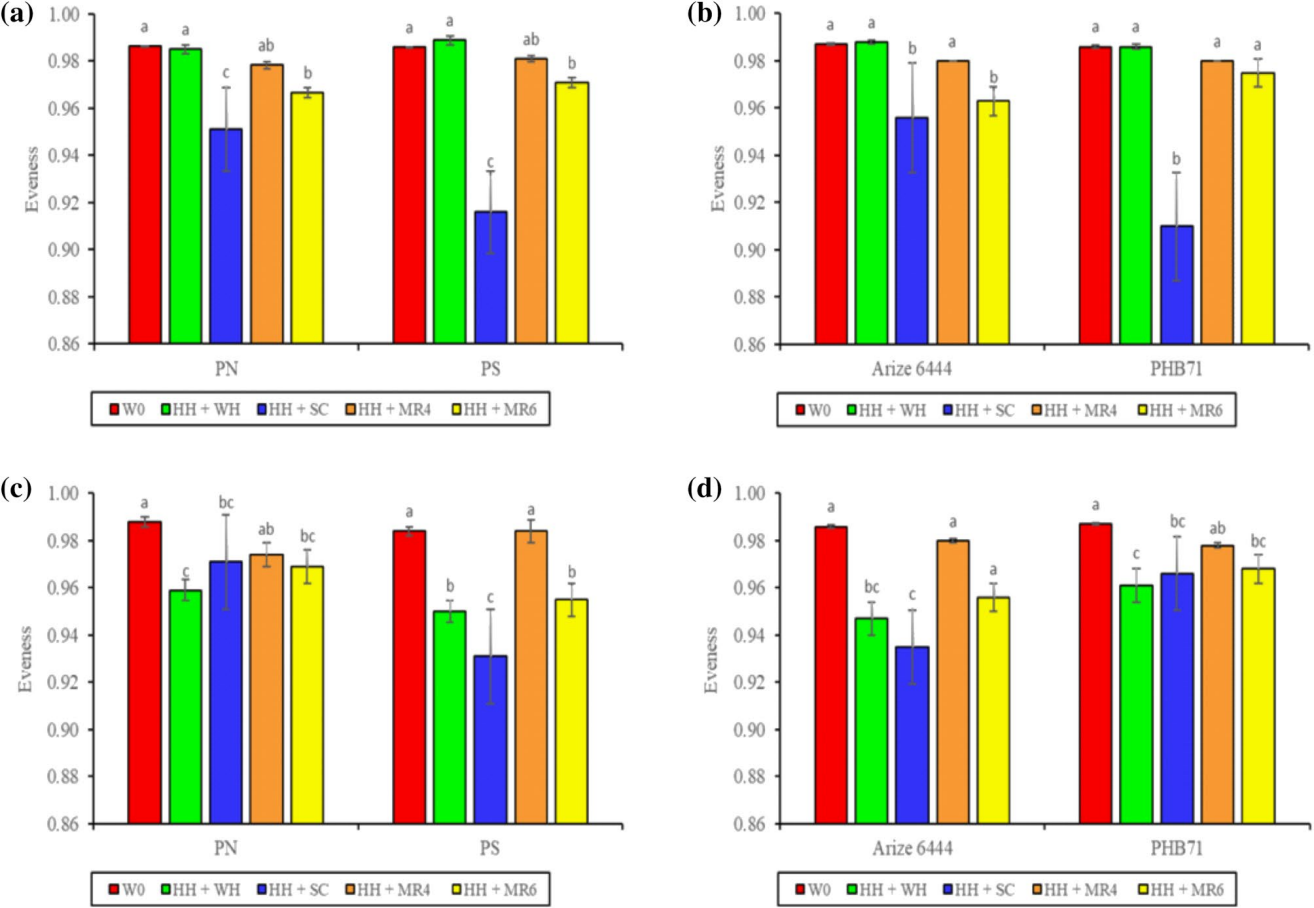
Interaction effect of planting geometry × weed management and cultivar × weed management on evenness at 25 (**a**,**b**) and 60 DAS (**c**,**d**). P_N_, sowing with seed drill at 18.5 cm row spacing; P_S_, square planting at 25 cm × 25 cm row to row and plant to plant spacing; W_C_, weedy check (no weed management); H_H_ + W_H_, one hand hoeing at 12 DAS *fb* one hand weeding at 30 DAS; H_H_ + S_C_, one hand hoeing at 12 DAS *fb Sesbania aculeata* co-culture and mulched 45 DAS; H_H_ + M_R4_, one hand hoeing at 12 DAS *fb* rice residue mulching @ 4 t ha^−1^; H_H_ + M_R6_, one hand hoeing at 12 DAS *fb* rice residue mulching @ 6 t ha^−1^. Means with different alphabets are significant (P < 0.05).

Weed diversity indices such as dominance, evenness, and diversity were not influenced by PG, CV, or their interaction (Table 2). However, weed management (WM) had a significant effect on all the weed indices at 25 and 60 DAS. The H_H_ + S_C_ weed management treatment had the lowest Shannon–Wiener and evenness indices but the highest dominance index (Table 2). The lowest Shannon–Wiener and evenness indices values in H_H_ + S_C_ treatment indicate greater control of weeds^42^. Data on evenness (close to 1) indicates that weed species distribution in this experiment is more uniform across treatments. Weed evenness was not influenced by the interaction between PG and CV and PG, CV and WM at either evaluation date (25 or 60 DAS). However, at 25 DAS, PG × WM and CV × WM had a significant effect on evenness (Fig. 2a,b).

**Table 2:** Effect of planting geometry, cultivar and non-chemical weed management on weed diversity in rice.

Treatment	25 DAS			60 DAS		
	Dominance	Evenness	Shannon–Wiener index	Dominance	Evenness	Shannon–Wiener index
Planting geometry (PG)						
P_N_	0.108 a*	0.974 a	2.25 a	0.106 a	0.973 a	2.27 a
P_S_	0.107 a	0.969 a	2.26 a	0.108 a	0.961 a	2.26 a
* P* value	0.6474	0.6128	0.1989	0.1642	0.1866	0.1730
Cultivar (CV)						
Arize 6444	0.108 a	0.975 a	2.25 a	0.108 a	0.961 a	2.26 a
PHB71	0.107 a	0.968 a	2.27 a	0.106 a	0.972 a	2.27 a
* P* value	0.5625	0.0851	0.1825	0.0531	0.0643	0.0532
Weed management (WM)						
W_C_	0.103 b	0.986 a	2.29 a	0.103 c	0.987 a	2.29 a
H_H_ + W_H_	0.103 b	0.987 a	2.29 a	0.110 a	0.955 bc	2.26 b
H_H_ + S_C_	0.124 a	0.934 c	2.16 b	0.110 a	0.951 c	2.25 c
H_H_ + M_R4_	0.104 b	0.980 a	2.28 a	0.104 b	0.980 a	2.28 a
H_H_ + M_R6_	0.106 b	0.969 b	2.27 a	0.107 c	0.962 b	2.26 b
* P* value	< 0.0001**	** < 0.0001**	** < 0.0001**	** < 0.0001**	** < 0.0001**	** < 0.0001**
*P* value (interaction)						
PG × CV	0.4221	0.7794	0.3800	0.5461	0.6926	0.6158
PG × WM	0.9991	**0.0005**	0.7732	**0.0002**	**0.0003**	**0.0004**
Cv × WM	0.9429	** < 0.0001**	0.1822	**0.0154**	**0.0130**	**0.0203**
PG × CV × WM	0.5600	0.7617	0.4509	0.6447	0.5209	0.4072
Year	0.4223	0.6974	0.3471	0.5176	0.4238	0.2987

*Means with different alphabets are significant (p < 0.05). DAS, days after sowing. P_N_, sowing with seed drill at 18.5 cm row spacing; P_S_, square planting at 25 cm × 25 cm row to row and plant to plant spacing; W_C_, weedy check (no weed management); H_H_ + W_H_, one hand hoeing at 12 DAS *fb* one hand weeding at 30 DAS; H_H_ + S_C_, one hand hoeing at 12 DAS *fb S. aculeata* co-culture and mulched 45 DAS; H_H_ + M_R4_, one hand hoeing at 12 DAS *fb* rice residue mulching @ 4 t ha^−1^; H_H_ + M_R6_, one hand hoeing at 12 DAS *fb* rice residue mulching @ 6 t ha^−1^.

**Bold P values are significant.

**Figure 3:**
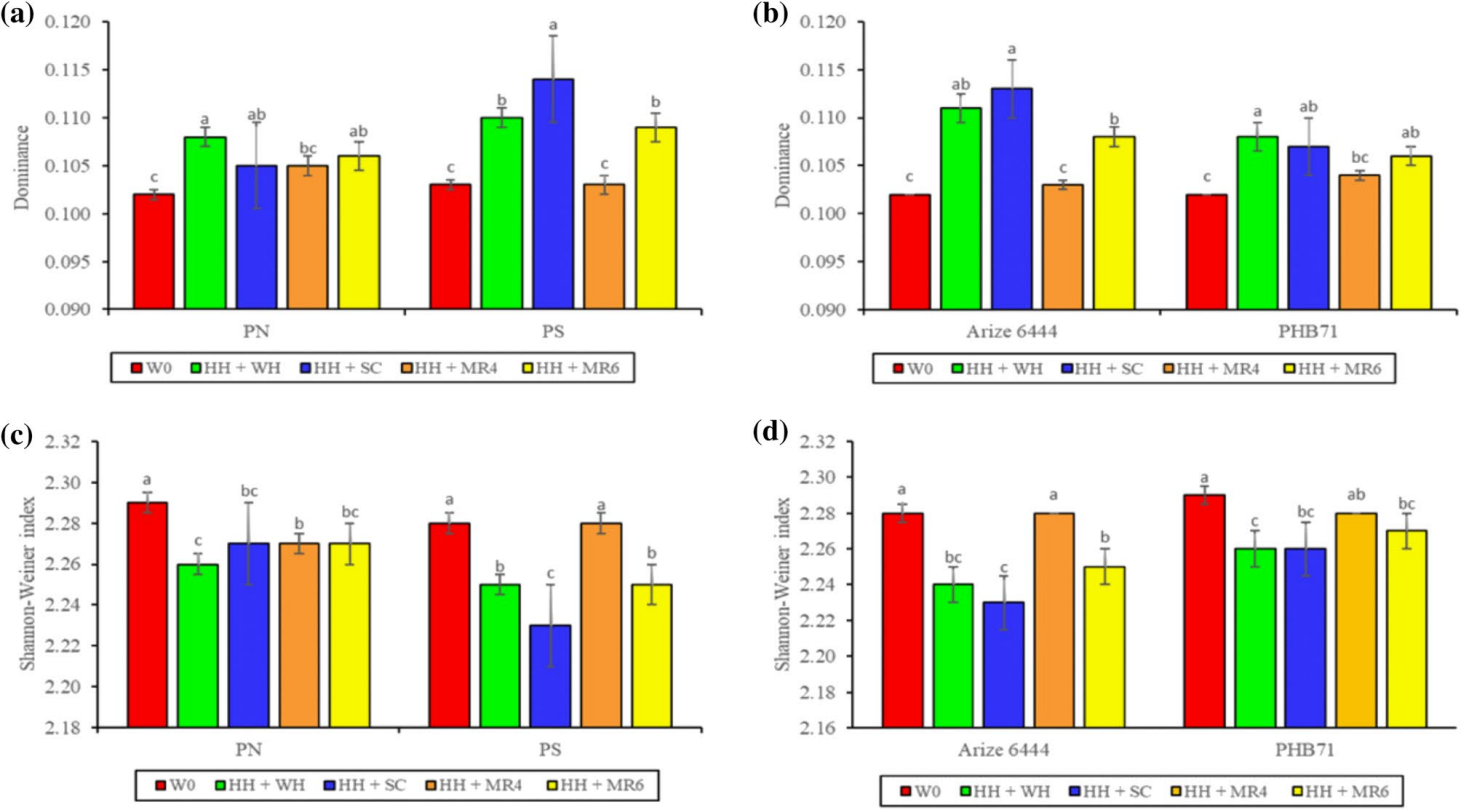
Interaction effect of planting geometry × weed management and cultivar × weed management on dominance (**a**,**b**) and Shannon-Weiner index (**c**,**d**) at 60 DAS. P_N_, sowing with seed drill at 18.5 cm row spacing; P_S_, square planting at 25 cm × 25 cm row to row and plant to plant spacing; W_C_, weedy check (no weed management); H_H_ + W_H_, one hand hoeing at 12 DAS *fb* one hand weeding at 30 DAS; H_H_ + S_C_, one hand hoeing at 12 DAS *fb Sesbania aculeata* co-culture and mulched 45 DAS; H_H_ + M_R4_, one hand hoeing at 12 DAS *fb* rice residue mulching @ 4 t ha^−1^; H_H_ + M_R6_, one hand hoeing at 12 DAS *fb* rice residue mulching @ 6 t ha^−1^. Means with different alphabets are significant (P < 0.05).

Likewise, at 60 DAS, the interaction of PG × WM and CV × WM had a significant effect on evenness (Fig. 2b,c), dominance (Fig. 3a,b), and the Shannon–Wiener index (Fig. 3b,c). Compared to other treatments, Ps and the H_H_ + S_C_ combination had significantly lower values of evenness and Shannon–Wiener index and the highest dominance value.

### Weed control efficiency indices

Planting geometry had a significant effect (p < 0.05) on WMI, AMI, and IWMI, but did not influence the CRI and WPI (p > 0.05, Table 3). Compared to the zero-till seed drill sown method, the square planting (P_S_) method had a lower WMI, AMI, and IWMI, which indicates the effectiveness of this method on weed suppression. The lowest values of WMI, AMI, and IWMI indicate better weed control and a higher yield. The lowest values of WMI and AMI were recorded in earlier studies by Mishra et al.^43^ and Kumar et al.^24^ with treatments that efficiently reduced weeds and increased grain yield.

**Table 3:** Effect of planting geometry, cultivar and non-chemical weed management on weed control efficiency indices in rice.

Treatment	WI (%)	CRI	WPI	WMI	AMI	IWMI
Planting geometry (PG)						
P_N_	17.90	1.94 a*	0.95 a	3.29 a	2.49 a	2.89 a
P_S_	15.46	1.98 a	0.93 a	1.70 b	0.90 b	1.30 b
* P* value		0.4937	0.1778	0.0332**	**0.0332**	**0.0332**
Cultivar (CV)						
Arize 6444	16.06	2.06 a	0.94 a	2.37 a	1.57 a	1.97 a
PHB71	17.30	1.85 b	0.94 a	2.62 a	1.82 a	2.22 a
* P* value		**0.0253**	0.6335	0.6719	0.6719	0.6719
Weed management (WM)						
W_C_	55.56	1.00 e	1.00 a	–	–	–
H_H_ + W_H_	0	2.69 a	0.90 d	2.15 d	1.15 d	1.65 d
H_H_ + S_C_	3.37	2.34 b	0.95 b	2.94 c	1.94 c	2.44 c
H_H_ + M_R4_	16.23	1.70 d	0.91d	4.00 a	3.00 a	3.50 a
H_H_ + M_R6_	8.24	2.06 c	0.93 c	3.40 b	2.40 b	2.90b
* P* value	–	** < 0.0001**	** < 0.0001**	** < 0.0001**	** < 0.0001**	** < 0.0001**
*P* value (Interaction)						
PG × CV	–	0.4704	0.9571	0.6862	0.6862	0.6862
PG × WM	–	0.9338	0.0412	** < 0.0001**	** < 0.0001**	** < 0.0001**
Cv × WM	–	**0.0393**	0.3985	0.4960	0.4960	0.4960
PG × CV × WM	–	0.7360	0.0692	0.7689	0.7689	0.7689
Year	–	0.7568	0.1994	0.8421	0.8267	0.7968

*Means with different alphabets are significant (p < 0.05). DAS, days after sowing. P_N_, sowing with seed drill at 18.5 cm row spacing; P_S_, square planting at 25 cm × 25 cm row to row and plant to plant spacing; W_C_, weedy check (no weed management); H_H_ + W_H_, one hand hoeing at 12 DAS *fb* one hand weeding at 30 DAS; H_H_ + S_C_, one hand hoeing at 12 DAS *fb S. aculeata* co-culture and mulched 45 DAS; H_H_ + M_R4_, one hand hoeing at 12 DAS *fb* rice residue mulching @ 4 t ha^−1^; H_H_ + M_R6_, one hand hoeing at 12 DAS *fb* rice residue mulching @ 6 t ha^−1^. WI, weed index; CRI, crop resistance index; WPI, weed persistence index; WMI, weed management index; AMI, agronomic management index; IWMI, integrated weed management index.

**Bold P values are significant.

Cultivars influenced the CRI significantly (p < 0.05) compared to other indices. The cultivar Arize 6444 resulted in a higher CRI value than the cultivar PHB 71. CRI indicates increased vigor of crop plants due to weed control. Superior crop growth and biomass production of the Arize 6444 cultivar could be the reason for the higher CRI value. Garko et al.^44^ also reported a significant effect of different varieties on CRI in maize crop. The weed management treatments greatly influenced all the weed management indices. Among weed management treatments, H_H_ + W_H_ performed well; therefore, this treatment had a 169% higher CRI than the weedy check.

Furthermore, H_H_ + W_H_ treatment resulted in the lowest values of WPI, WMI, AMI, and IWMI over other treatments. The lower WPI, WMI, AMI, and IWMI indicate superior weed control.

The interaction effect of planting geometry × weed management revealed that P_S_ × H_H_ + W_H_ had a lower value of WPI, WMI, AMI, and IWMI (Fig. S2a). Square planting and hand hoeing at the early stage *fb* hand weeding at peak weed emergence period could have resulted in better weed control than other combinations. The interaction of cultivar × weed management only had a significant effect on CRI (Fig. S2b). Greater suppression and control of weeds under the combination of Arize 6444 × H_H_ + W_H_ treatment might have led to a higher CRI.

### Crop growth parameters

Planting geometry, cultivar, and weed management influenced the crop growth parameters (Table 4). However, the interaction effect of planting geometry, cultivar, and weed management did not influence except the number of tillers by planting geometry-by-cultivar and dry matter production by cultivar-by-weed management. The number of tillers (number m^−2^) and dry matter production (g running m^−1^) were (p < 0.05) 7.6% and 13.11% higher, respectively, for the square planting (P_S_) compared to the zero-till seed drill sown crop (P_N_). This could be due to optimum crop spacing that allowed the radiant energy, nutrients, and water to utilize; as a result, more tillers and robust crop growth were achieved under the square planting method^6^. On the other hand, De Datta^45^, reported that a higher seed rate in a seed drill-sown crop with normal spacing increases inter-and intra-plant competition, which leads to poor utilization of applied inputs, poor crop growth, and a lesser number of tillers. Furthermore, the square planting treatment decreased weed competition compared to zero-till seed drill-sown crop; this could also be the reason for the better growth and development. Cultivars only influenced the dry matter accumulation but not the number of tillers (Table 4). The Arize 6444 resulted in 8.41% higher dry matter production than the PHB 71; the higher dry matter for Arize 6444 could be the result of greater plants height and tiller production^46^.

**Table 4:** Effect of planting geometry, cultivar and non-chemical weed management on growth attributes and yield in rice.

Treatment	Tiller number (m^−2^)	Dry matter (g running m^−1^)	Grain yield (t ha^−1^)	Production efficiency (kg ha^−1^ day^−1^)
Planting geometry (PG)				
P_N_	316.0 b*	89.48 b	3.89 b	30.89 b
P_S_	342.0 a	102.98 a	4.19 a	33.22 a
* P* value	0.0165**	**0.0407**	**0.0295**	**0.0295**
Cultivar (CV)				
Arize 6444	341.9 a	100.46 a	4.24 a	33.68 a
PHB71	316.1 a	92.01 b	3.83 b	30.43 b
* P* value	0.0820	**0.0182**	**0.0035**	**0.0035**
Weed management (WM)				
W_C_	196.7 d	68.07 d	2.16 d	17.13 d
H_H_ + W_H_	378.7 a	110.18 a	4.85 a	38.45 a
H_H_ + S_C_	371.8 a	108.78 a	4.68 a	37.16 a
H_H_ + M_R4_	338.1 c	91.74 c	4.05 c	32.16 c
H_H_ + M_R6_	359.6 b	102.41 b	4.46 b	35.37 b
*P* value	** < 0.0001**	** < 0.0001**	** < 0.0001**	** < 0.0001**
*P* value (Interaction)				
PG × CV	0.8504	0.9517	0.0968	0.0967
PG × WM	**0.0120**	0.9765	**0.0210**	**0.0211**
Cv × WM	0.9636	**0.0350**	**0.0246**	**0.0245**
PG × CV × WM	0.9962	0.8571	0.9297	0.9303
Year	0.7256	0.5214	0.6217	0.3327

*Means with different alphabets are significant (p < 0.05). DAS, days after sowing. P_N_, sowing with seed drill at 18.5 cm row spacing; P_S_, square planting at 25 cm × 25 cm row to row and plant to plant spacing; W_C_, weedy check (no weed management); H_H_ + W_H_, one hand hoeing at 12 DAS *fb* one hand weeding at 30 DAS; H_H_ + S_C_, one hand hoeing at 12 DAS *fb S. aculeata* co-culture and mulched 45 DAS; H_H_ + M_R4_, one hand hoeing at 12 DAS *fb* rice residue mulching @ 4 t ha^−1^; H_H_ + M_R6_, one hand hoeing at 12 DAS *fb* rice residue mulching @ 6 t ha^−1^.

**Bold P values are significant.

The weed management treatments, H_H_ + W_H_ and H_H_ + S_C_ resulted in the highest number of tillers and dry matter compared to other weed management treatments (Table 4). Hoeing and hand weeding at the early phases of crop growth might have nullified the early weed competition and ultimately led to a greater number of tillers and dry matter. Our findings are in agreement with Johnson et al.^47^ who reported that early-stage weed control in direct-seeded rice reduced weed pressure and increased grain yield. Growing *S. aculeata* and retaining its mulch in rice can suppress the weeds effectively^48^. Additionally, mineralization of residues provides available nutrients to crops at critical stages, which has a positive effect on crop growth at an early stage^49,50^.

**Figure 4:**
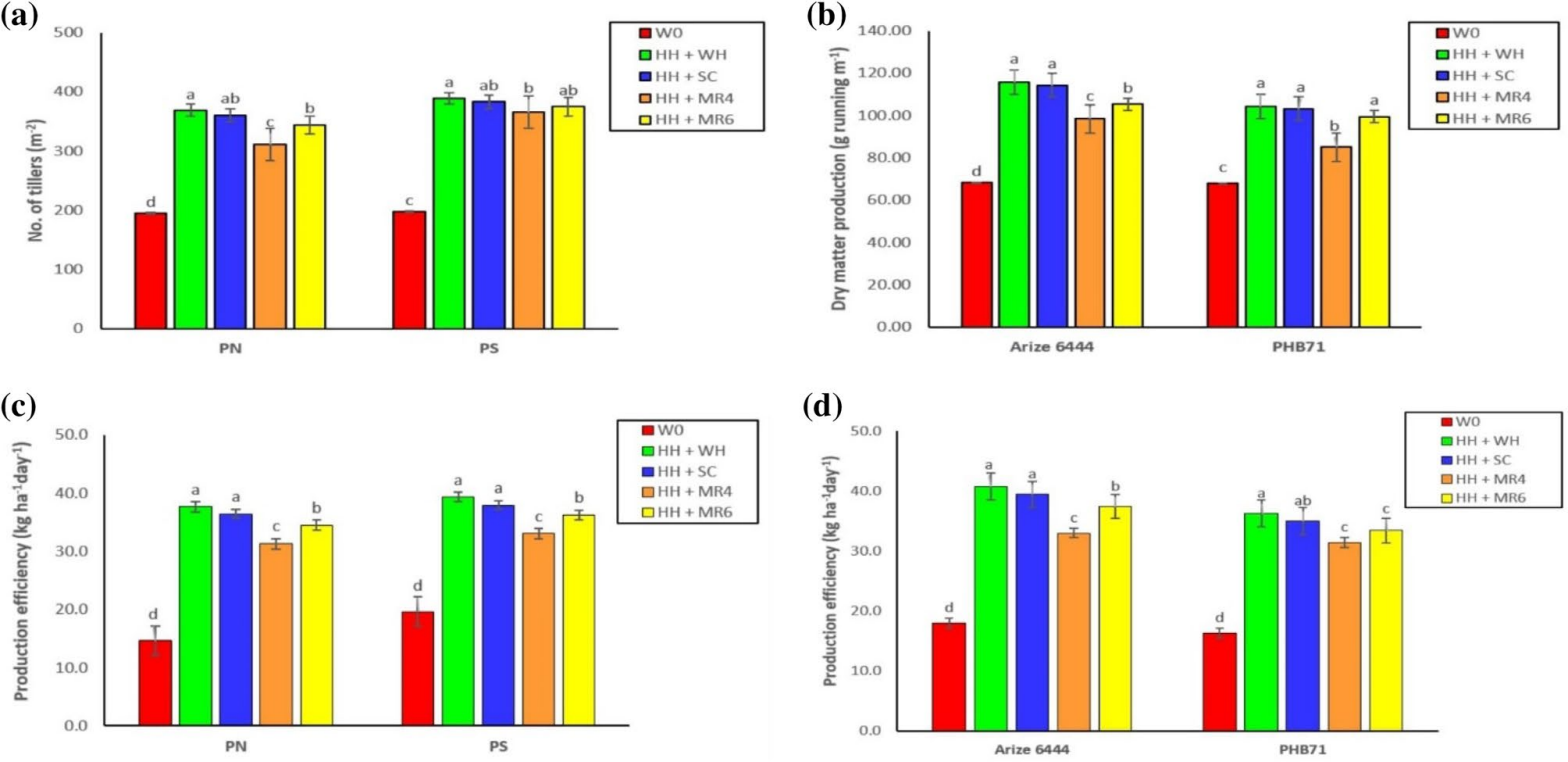
Interaction effect of planting geometry × weed management, and cultivar × non-chemical weed management on number of tillers (**a**), dry matter production (**b**) and production efficiency of rice (**c**,**d**). P_N_, sowing with seed drill at 18.5 cm row spacing; P_S_, square planting at 25 cm × 25 cm row to row and plant to plant spacing; W_C_, weedy check (no weed management); H_H_ + W_H_, one hand hoeing at 12 DAS *fb* one hand weeding at 30 DAS; H_H_ + S_C_, one hand hoeing at 12 DAS *fb Sesbania aculeata* co-culture and mulched 45 DAS; H_H_ + M_R4_, one hand hoeing at 12 DAS *fb* rice residue mulching @ 4 t ha^−1^; H_H_ + M_R6_, one hand hoeing at 12 DAS *fb* rice residue mulching @ 6 t ha^−1^. Means with different alphabets are significant (P < 0.05).

Planting geometry × weed management had a significant effect on the number of tillers. The interaction effect of P_S_ and H_H_ + W_H_ resulted in a maximum number of tillers, followed by P_N_ and H_H_ + S_C_ and P_N_ and H_H_ + M_R_ treatment combinations (Fig. 4a). Cultivar × weed management was found significant for dry matter accumulation. Arize 6444 and H_H_ + W_H_, Arize 6444 and H_H_ + S_C_ combinations had a higher dry matter accumulation (Fig. 4b). The authors believed that this could be due to the synergistic effect of wider spacing in square planting and control of weeds by hand hoeing *fb* hand weeding, and hand hoeing *fb Sesbania* co-culture treatments.

### Crop productivity

Compared to zero-till seed drill-sown crop (P_N_), square planting (P_S_) achieved a ~ 7.6% higher grain yield (Table 4). Vigorous crop growth, minimum inter-specific competition, a higher number of tillers, and greater weed suppression might be responsible for higher yields in the square planting method^12,18,51^. Previous studies reported that direct-seeded rice in the square planting method had a higher grain yield compared to normal planting^52^. Among cultivars, Arize 6444 produced a 10.7% higher grain yield than PHB 71. The higher grain yield for Arize 6444 could be attributed to increased dry matter accumulation, more tillers, faster crop growth, and better weed suppression^15^. With respect to weed management tactics, the H_H_ + W_H_ recorded the highest grain yields (4.85 t ha^−1^), followed by the H_H_ + S_C_ (4.68 t ha^−1^). Hoeing *fb* hand weeding during the critical crop-weed competition period might have reduced the weed competition and led to better crop performance^14,53^. Early weed control is crucial in DSR for improved crop growth and yield^5,6^. The hand hoeing *fb* either hand weeding or *Sesbania* spp. co-culture resulted in a weed-free condition and improved yield. Similarly, Maity and Mukherjee^49^, also reported that co-culture of *Sesbania* with rice smothered weeds and enhanced the grain yield of rice. Likewise, Baumann et al.^27^, and Gopal et al.^54^, observed a higher grain yield and available N content in soil under *S. aculeata* co-culture in direct seeding.

The interaction between planting geometry or cultivar and weed management tactics significantly influenced the grain yield. The treatment P_S_ and H_H_ + W_H_ combination achieved the highest grain yield, followed by P_S_ and H_H_ + S_c_, P_N_ and H_H_ + S_c_, and P_N_ and H_H_ + W_H_ (Table S1). Among weed management and cultivar interactions, the highest grain yield was observed for Arize 6444 × H_H_ + W_H_ and Arize 6444 × H_H_ + Sc combinations (Table S2). Better crop growth, higher dry matter accumulation, and greater weed suppression ability of the Arize 6444 cultivar with square planting and hand hoeing *fb* hand weeding or hand hoeing *fb Sesbania* spp. co-culture could be the reasons for the higher grain yield.

Planting geometry, cultivar, and weed management had a significant impact on production efficiency. The results showed that square planting (P_S_) had the maximum production efficiency compared to zero-till seed drill-sown crops (P_N_). The higher yield with square planting could be attributed to improved production efficiency. Among cultivars, Arize 6444 resulted in higher production efficiency than PHB 71. The H_H_ + W_H_ achieved the highest production efficiency across weed management treatments and was comparable to H_H_ + Sc. The increased crop yield per day was believed to be a reason for the higher production efficiency in H_H_ + W_H_ and H_H_ + Sc. The P_S_ and H_H_ + W_H_ interactions increased production efficiency (Fig. 4c). The cultivar × weed management interaction revealed that maximum production efficiency was recorded for Arize 6444 and H_H_ + W_H_ (Fig. 4d). This could also be because of the higher grain yield ha^−1^ day^−1^ and effective weed control^12^.

### Economic analysis

**Figure 5:**
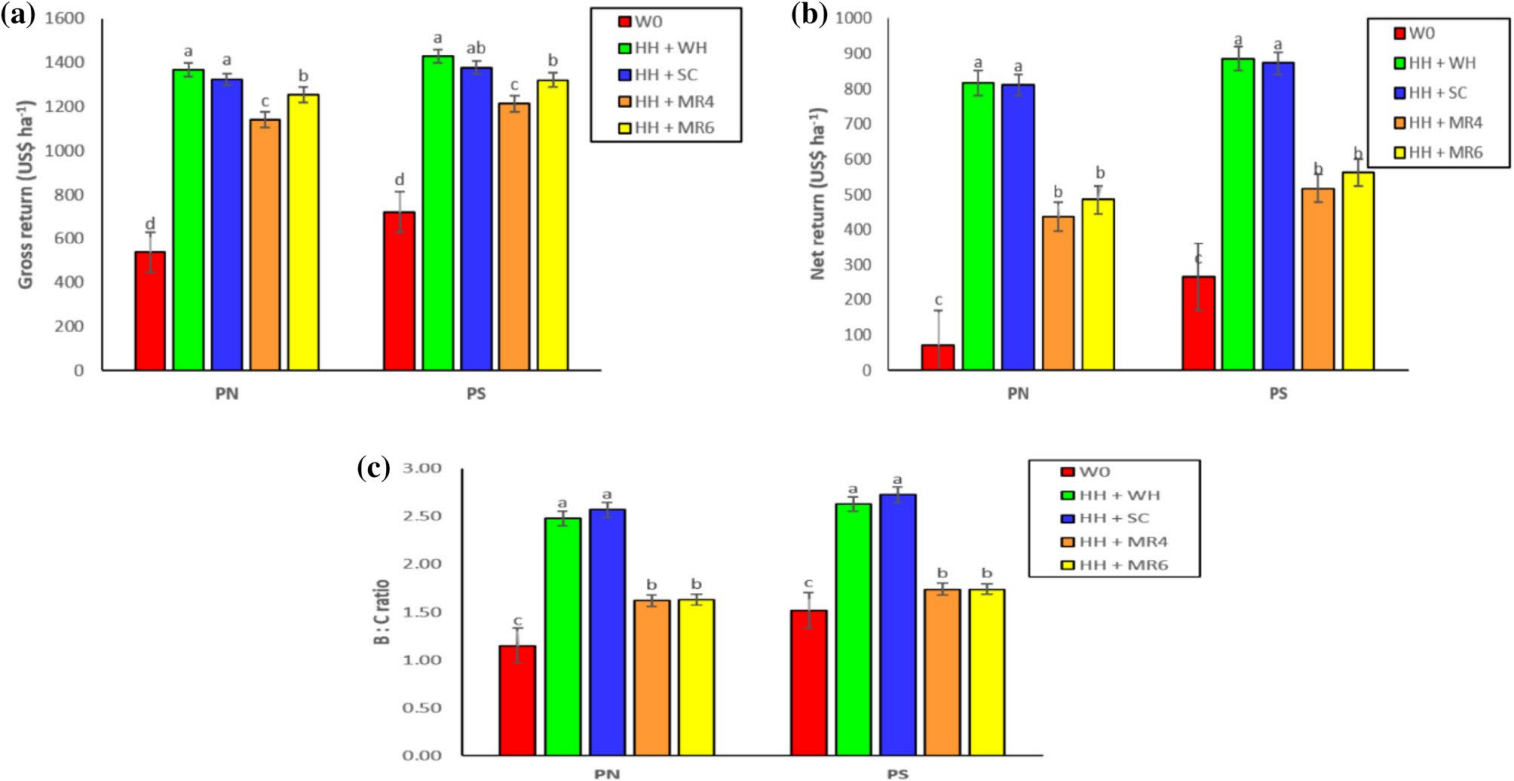
Interaction effect of planting geometry × weed management on gross return, net return and B:C ratio of rice. P_N_, sowing with seed drill at 18.5 cm row spacing; P_S_, square planting at 25 cm × 25 cm row to row and plant to plant spacing; W_C_, weedy check (no weed management); H_H_ + W_H_, one hand hoeing at 12 DAS *fb* one hand weeding at 30 DAS; H_H_ + S_C_, one hand hoeing at 12 DAS *fb Sesbania aculeata* co-culture and mulched 45 DAS; H_H_ + M_R4_, one hand hoeing at 12 DAS *fb* rice residue mulching @ 4 t ha^−1^; H_H_ + M_R6_, one hand hoeing at 12 DAS *fb* rice residue mulching @ 6 t ha^−1^. Means with different alphabets are significant (P < 0.05).

A slightly higher cost of cultivation (COC) was registered for P_N_ (600.55 US$) compared to P_S_ (591.47 US$), which was due to the higher cost of hybrid seeds used under seed-drill sown crops (Table 5). Square planting had higher gross returns (GR) by 7.24%, net returns (NR) by 15.59%, and B: C ratio of 8.78% than zero-till drill sown crops. This was because of the lower COC coupled with a higher GR in P_S_ than P_N_. Cultivars did not influence the COC due to similar seed rates, seed costs, and other inputs. However, higher GR, NR, and BCR were obtained for Arize 6444 compared to PHB 71 because of the higher yield under Arize 6444^12,18^. The COC for weed control treatments ranged from 459.46 to 763.22 US$ ha^−1^; W_C_ had the lowest COC and H_H_ + M_R6_ had the highest. Rice straw was applied at a rate of 6 t ha^−1^ and the higher cost of rice straw was the reason for the higher COC in the H_H_ + M_R6_ treatment. Higher GR (1398.26 US$ ha^–1^) and NR (851.03 US$ ha^–1^) were observed under H_H_ + W_H_ and H_H_ + S_C,_ and the least was with W_C_ in both years. However, BCR was higher for H_H_ + S_C_ *fb* H_H_ + W_H_ treatment. These results could be the result of lower weed density under H_H_ + W_H_ and H_H_ + S_C_^14^. The interaction effect between planting geometry × weed management was found to be significant for GR, NR and BCR (Fig. 5a–c). Highest GR, NR, and BCR were recorded under interaction of P_s_ and H_H_ + W_H_ as compared to other treatment combinations.

**Table 5:** Effect of planting geometry, cultivar and non-chemical weed management on cost of cultivation, gross return, net return and B: C ratio.

Treatment	Cost of cultivation (US$ ha^−1^)	Gross return (US$ ha^−1^)	Net return (US$ ha^−1^)	Benefit: cost ratio
Planting geometry (PG)				
P_N_	600.55	1124.69 b*	524.14 b	1.87 b
P_S_	591.47	1212.45 a	620.98 a	2.05 a
* P* value	–	0.0355**	**0.0373**	**0.0200**
Cultivar (CV)				
Arize 6444	594.44	1225.14 a	630.70 a	2.06 a
PHB71	594.44	1112.00 b	517.56 b	1.87 b
* P* value	–	**0.0050**	**0.0045**	**0.0043**
Weed management (WM)				
W_C_	459.46	627.80 d	168.34 c	1.37 c
H_H_ + W_H_	547.22	1398.26 a	851.03 a	2.56 a
H_H_ + S_C_	510.15	1352.32 a	842.17 a	2.65 a
H_H_ + M_R4_	700.00	1176.99 c	477.00 b	1.68 b
H_H_ + M_R6_	763.22	1287.48 b	524.26 b	1.69 b
* P* value	–	** < 0.0001**	** < 0.0001**	** < 0.0001**
*P* value (Interaction)				
PG × CV	–	0.1080	0.1165	0.1287
PG × WM	–	**0.0423**	**0.0423**	**0.0026**
Cv × WM	–	0.1565	0.1564	0.0791
PG × CV × WM	–	0.9753	0.9752	0.9939
Year	–	0.8717	0.8810	0.8623

*Means with different alphabets are significant (p < 0.05). DAS, days after sowing. P_N_, sowing with seed drill at 18.5 cm row spacing; P_S_, square planting at 25 cm × 25 cm row to row and plant to plant spacing; W_C_, weedy check (no weed management); H_H_ + W_H_, one hand hoeing at 12 DAS *fb* one hand weeding at 30 DAS; H_H_ + S_C_, one hand hoeing at 12 DAS *fb S. aculeata* co-culture and mulched 45 DAS; H_H_ + M_R4_, one hand hoeing at 12 DAS *fb* rice residue mulching @ 4 t ha^−1^; H_H_ + M_R6_, one hand hoeing at 12 DAS *fb* rice residue mulching @ 6 t ha^−1^.

**Bold P values are significant.

## Conclusions

The results emphasize the importance of selecting appropriate weed management strategies for sustainable DSR, taking into account both environmental considerations and economic feasibility. The findings from this study revealed that Arize 6444, the square planting system, and the hoeing *fb* hand weeding performed better in terms of yield than PHB 71, normal planting and other weed management practices. However, the higher cost of manual weeding and the unavailability of labors are the main drawbacks of the hoeing *fb* hand weeding system. Alternatively, Arize 6444, square planting geometry, and hoeing at 12 DAS *fb Sesbania* co-culture mulch at 45 DAS enhanced the productivity and profitability of DSR and significantly reduced weed density in the Eastern region of India. These findings contribute valuable insights to the ongoing efforts to promote sustainable and environmental friendly weed management practices, mitigating the risks associated with herbicide use and potential evolution of resistant weeds in direct-seeded rice systems. Development and research on precise seeding machines is a future research area for wider adoption of hybrids in DSR systems, higher weed control efficiency, and higher yield. Additionally, an assessment of the long-term impacts of the proposed weed management strategies on soil health, biodiversity, and overall ecosystem resilience is needed.

## Materials and methods

### Experimental site and weather conditions

Field experiments were carried out at the Agricultural Research Farm of the Institute of Agricultural Sciences, Banaras Hindu University, Varanasi (25,018′ N and 88,003′E), Uttar Pradesh, India, during the rainy seasons (June to October) in 2015 and 2016. The cropping system at the site has been rice followed by wheat for the last six years. The climate of the site is sub-tropical; May and June were the hottest months (maximum temperature 31–36 °C) and January was the coldest month (minimum temperature 7–14 °C). Annual rainfall averages 1036.8 mm and 87.3% of them are received between June and September (South-West Monsoon), and the remaining 13.7% is received between October and May (western disturbances and other climatological factors). The weather parameters are presented in Fig. S3. The soil type was a sandy clay loam (Typic Haplusteptiso-hyperthermic family, Inceptisol)^55^ with 0.4% organic carbon, 7.5 pH, 0.21 dsm^–1^ EC, 182.67 kg ha^–1^ available N, 22.12 kg ha^–1^ available P, and 216.5 kg ha^–1^ exchangeable K.

### Treatment details and crop management

The experiments were arranged in a split-split plot design with three trial factors (planting geometries, cultivars, and non-chemical weed management) in three replications. Two planting geometries [normal (PN) and square planting (PS)] were arranged in the main-plots, two cultivars (Arize 6444 and PHB 71) in the sub-plots, and five non-chemical weed management treatments [weedy check (WC), single hoeing (1 HH) at 12 DAS fb one hand weeding (1 HW) at 30 DAS (HH + WH), 1 HH at 12 DAS fb Sesbania co-culture and its mulching (HH + Sc), 1 HH at 12 DAS fb rice straw mulching @ 4t ha^−1^ (HH + MR4), and 1 HH at 12 DAS rice straw mulching @ 6 t ha^−1^ (HH + MR6)] in the sub-subplots (Table 1). The main plot size was 40 m × 5 m, the sub-plot size was 20 m × 5 m, and the sub-sub plot was 4 m × 5 m. The field was prepared with one pass of moldboard plough fb disk to uproot established perennial weeds. Finally, two passes of cultivator and planking were done to provide a good tilth suitable for a DSR crop. The sowing dates were June 22, in 2015 and June 28, in 2016. Nitrogen (150 kg ha^−1^), P_2_O_5_ (60 kg ha^−1^), and K_2_O (60 kg ha^−1^) were applied at the recommended rates through urea (NH_2_)_2_CO), di-ammonium phosphate ((NH4)_2_HPO_4_) and muriate of potash (KCl), respectively. Half of the recommended nitrogen and full doses of phosphorus and potassium were applied at the time of sowing. The remaining nitrogen was given in two equal portions at the tillering and panicle initiation stages. The crop was harvested manually on 28th October in 2015 and 5th November in 2016 (Table 6).

**Table 6:** Description of planting geometry, cultivar and weed management options adopted in the experiment.

S. no	Abbreviation	Rice establishment/weed management
1	P_N_	Rice was sown using 30 kg seed ha^−1^ by tractor-drawn zero till seed drill at a row spacing of 18.5 cm apart
2	P_S_	Rice was sown using 12 kg seed ha^−1^ by *kudal* (local furrow maker) manually at 25 cm × 25 cm row to row and plant to plant spacing
3	Arize 6444	Hybrid of medium duration (135–140 days), medium slender grain, high productive tillers, more grains panicle^−1^ (250–300), wider adaptability, more than 70% milling
4	PHB71	Hybrid, medium duration (130–135 days), tall (130 cm), non-shattering, long slender grains, high milling (71%)
4	W_C_	Weedy check (Full season weed competition)
5	H_H_ + W_H_	one hand hoeing was done at 12 DAS *fb* one hand weeding at 30 days after sowing
6	H_H_ + Sc	one hand hoeing was done at 12 DAS *fb Sesbania aculeate* co-culture (*Sesbania aculeata* was sown in between rice rows manually by using 25 kg seed ha^−1^). After that *Sesbania aculeata* was harvested manually with the help of sickle at 45 DAS and green residue was placed in between rice rows
7	H_H_ + M_R4_	one hand hoeing was done at 12 DAS *fb* rice straw mulching @4 t ha^−1^. Rice straw of last year crop was weighed and spread uniformly just after hoeing in between rice rows
8	H_H_ + M_R6_	one hand hoeing was done at 12 DAS *fb* rice straw mulching @6 t ha^−1^. Rice straw of last year crop was weighed and spread uniformly in between rice rows just after hoeing

### Weed observations

#### Weed density and composition

In each plot, two quadrates (1 m^2^) were placed randomly for weed observations (25 and 60 DAS). Weeds were classified as grass, broadleaf, and sedge after identification. At 60 DAS, the relative density of various weed flora was calculated by dividing the weed density of each weed species by the overall weed density in the weedy check plot and multiplying the result by 100.

#### Weed diversity indices

Weed dominance, diversity and evenness were assessed at 25 and 60 DAS by estimating the Simpson’s index^56^, Shannon–Wiener index^57^ and Pielou’s measure^58^, respectively using the Past software (v.4.03) (Eqs. 1–3).

1 $$\mathrm{Simpsons index }({\text{D}})=\sum \frac{(ni (ni-1))}{(N (N-1))}$$

2 $$ {\text{Shannon}}{-}{\text{Wiener index}}\;\left( {\text{H}} \right) = - \sum pi{\text{In}}pi $$

where ni is the number of species *i*, *pi* is the proportion of the species *i* in total number of species, N is the total number of individuals in a sample.

3 $$ {\text{Pielous}}\;{\text{measure}}\;{\text{of}}\;{\text{evenness}}\;\left( {\text{E}} \right) = {\text{H}}/{\text{In}}\;{\text{S}} $$

where H is the species diversity index (i.e., Shannon–Wiener index), and S is the species richness (number of weed species present in a plot).

#### Weed control indices

The weed control efficiency indices were calculated using weed dry matter and density data as well as crop dry matter and yield data at 25 and 60 DAS^59,60^ (Eqs. 4–8).

4 $${\text{CRI}}=\frac{{{\text{DMC}}}_{{\text{T}}}}{{\text{DMC}}_{\text{C}}}\times \frac{{\text{DMW}}_{\text{C}}}{{\text{DMW}}_{\text{T}}}$$

where, CRI = Crop Resistance Index, DMC_T_ = Dry matter of crop in treated plot, DMC_C_ = Dry matter of crop in control plot (weedy), DMW_C_ = Dry matter of weed in control plot, DMW_T_ = Dry matter of weed in treated plot.

5 $${\text{WPI}}=\frac{{{\text{DMW}}}_{{\text{T}}}}{{\text{DMW}}_{\text{C}}}\times \frac{{\text{WC}}_{\text{C}}}{{\text{WC}}_{\text{T}}}$$

where, WPI = Weed Persistence Index, DMW_T_ = Dry matter of weed in treated plot, DMW_C_ = Dry matter of weed in control plot, WC_C_ = Weed count in control plot, WC_T_ = Weed count in treated plot.

6 $${\text{WMI}}=\frac{\frac{{{\text{Y}}}_{{\text{T}}}-{{\text{Y}}}_{{\text{C}}}}{{{\text{Y}}}_{{\text{C}}}}}{\frac{{{\text{DMW}}}_{{\text{C}}}-{{\text{DMW}}}_{{\text{T}}}}{{{\text{DMW}}}_{{\text{C}}}}}$$

where, WMI = Weed Management Index, Y_T_ = Crop yield in treated plot, Y_C_ = Crop yield in control plot, DMW_C_ = Dry matter of weed in control plot, DMW_T_ = Dry matter of weed in treated plot.

7 $${\text{AMI}}=\frac{\frac{{{\text{Y}}}_{{\text{T}}}-{{\text{Y}}}_{{\text{C}}}}{{{\text{Y}}}_{{\text{C}}}}-\frac{{{\text{DMW}}}_{{\text{C}}}-{{\text{DMW}}}_{{\text{T}}}}{{{\text{DMW}}}_{{\text{C}}}}}{\frac{{{\text{DMW}}}_{{\text{C}}}-{{\text{DMW}}}_{{\text{T}}}}{{{\text{DMW}}}_{{\text{C}}}}}$$

where, AMI = Agronomic Management Index, Y_T_ = Crop yield in treated plot, Y_C_ = Crop yield in control plot, DMW_C_ = Dry matter of weed in control plot, DMW_T_ = Dry matter of weed in treated plot.

8 $${\text{IWMI}}=\frac{{\text{WMI}}+{\text{AMI}}}{2}$$

where, IWMI = Integrated Weed Management Index, WMI = Weed Management Index, AMI = Agronomic Management Index.

### Crop studies

At 90 DAS, the number of tillers in each plot was counted from a 1 m^2^ area. To calculate the dry matter accumulation, destructive plant sampling was performed from a meter row. These samples were sun-dried and then oven-dried at 65 °C for 72 h to achieve a constant dry weight. The plant dry weight is expressed in g m^−1^ row length. At harvest, plot-wise produce was threshed independently, and grain yield was measured in kg ha^–1^. The production efficiency was calculated (kg ha^−1^ day^−1^) by dividing the grain yield by the number of days needed for each treatment to reach maturity.

### Economic analysis

The economics were computed using current market input prices and the return on the final output (grain and straw yield). The production cost includes human labour, tilling, seeding, seed, straw, fertilizer, irrigation, harvesting and threshing, and the cost of transportation to market (Table S3). The following formulae were used to calculate the gross and net returns and the benefit-cost (B: C) ratio (Eqs. 9–11)^24^.

9 $$ {\text{Gross}}\;{\text{returns}}\;\left( {{\text{US}}\$ \;{\text{ha}}^{ - 1} } \right) = ({\text{Grain}}\;{\text{yield}}\;\left( {{\text{t}}\;{\text{ha}}^{ - 1} } \right) \times {\text{sale}}\;{\text{price}}\;\left( {{\text{US}}\$ \;{\text{t}}^{ - 1} } \right) + ({\text{Straw}}\;{\text{yield}}\;\left( {{\text{t}}\;{\text{ha}}^{ - 1} } \right) \times {\text{sale}}\;{\text{price}}\;\left( {{\text{US}}\$ \;{\text{t}}^{ - 1} } \right) $$

10 $$ {\text{Net}}\;{\text{return}}\;\left( {{\text{US}}\$ \;{\text{ha}}^{ - 1} } \right) = {\text{Gross}}\;{\text{returns}}\;\left( {{\text{US}}\$ \;{\text{ha}}^{ - 1} } \right){-}{\text{Cost}}\;{\text{of}}\;{\text{cultivation}}\;\left( {{\text{US}}\$ \;{\text{ha}}^{ - 1} } \right) $$

11 $$ {\text{Benefit}}\;{\text{Cost}}\;{\text{ratio}} = {\text{Gross}}\;{\text{return}}\;\left( {{\text{US}}\$ \;{\text{ha}}^{ - 1} } \right)/{\text{Cost}}\;{\text{of}}\;{\text{cultivation}}\;\left( {{\text{US}}\$ \;{\text{ha}}^{ - 1} } \right) $$

All economic analyses were expressed in US$ by converting 1 USD = 67 Indian rupees (INR).

### Statistical analysis

The data were subjected to analysis of variance (ANOVA) as described by Gomez and Gomez^61^. The normality of weed data was confirmed using the Shapiro–Wilk test (p < 0.05) and it was found non-normal. Therefore, the square-root transformation √(x + 0.5) was performed. Weed diversity indices such as dominance, diversity and evenness were calculated using the PAST software (version 4.03). In ANOVA, planting geometry, cultivars, weed management, and year were considered as the fixed effects, and replication was considered as the random effect. We did the combine analysis of data and found that there was no significant effect (p > 0.05) of years on weed density, diversity indices, weed control efficiency indices, available NPK in soil, number of tillers, dry matter production, grain yield, production efficiency and economics. Therefore, we did the pooled analysis of years. The treatment means were compared using Fisher’s LSD test at a 5% level of significance. All the analysis was performed using R software (version 4.0)^62^.

Authors have confirmed that all the plant studies were carried out in accordance with relevant national, international or institutional guidelines.

### Supplementary Information

Supplementary Information.

## Data Availability

The data that support the findings of this study are available on request from the corresponding author. The data are not publicly available due to private and ethical restrictions.
