# Supplementary material for: Ecological weed management and square planting influenced the weed management, and crop productivity in direct-seeded rice
Source: Sci Rep. 2024 May 6;14:10356. doi: 10.1038/s41598-024-56945-y (PMC11074338; doi:10.1038/s41598-024-56945-y)
Supplement: Supplementary file 1 — Supplementary Information. [file 41598_2024_56945_MOESM1_ESM.docx]

Ecological weed management and square planting influenced the weed management, and crop productivity in direct-seeded rice

**Mona Nagargade^1,2#^, Manoj Kumar Singh^2#^, Vishal Tyagi^1^*^#^, Prabhu Govindasamy^1,3^*^#^, Anil K. Choudhary^4^, Kuldeep Rajpoot^2^, Adarsh Kumar^5^, Preeti Singh^6^ & Debalin Sarangi^7^***

^1^Agronomy, ICAR-Indian Agricultural Research Institute, New Delhi, 110012, India,

^2^ Institute of Agricultural Sciences, Banaras Hindu University, Varanasi, Uttar Pradesh, 221005, India,

^3^ ICAR-National Research Centre for Banana, Tiruchirappalli, 620 102, India,

^4^ ICAR-Central Potato Research Institute, Shimla, Himachal Pradesh, 171001, India,

^5^ICAR-National Bureau of Agriculturally Important Microorganisms, Mau, Uttar Pradesh, 275101, India,

^6^ ICAR- Indian Agricultural Research Institute, Jharkhand, 825405, India

^7^University of Minnesota, MN 55108-6026, USA

#equally contributed

**Figure legends**

**Figure S1.** Relative density (%) of different weed species.

**Figure S2.** Interaction effect of planting geometry × weed management on WPI, WMI, AMI, IWMI (a) and cultivar × weed management on CRI (b) in rice.

**Figure S3.** Monthly rainfall (total), mean maximum and mean minimum temperature during crop season.


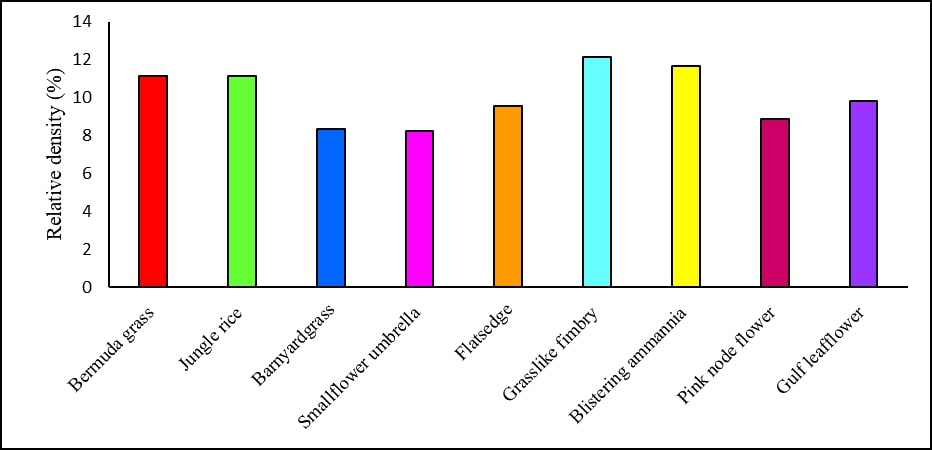


**Figure S1.** Relative density (%) of different weed species

**
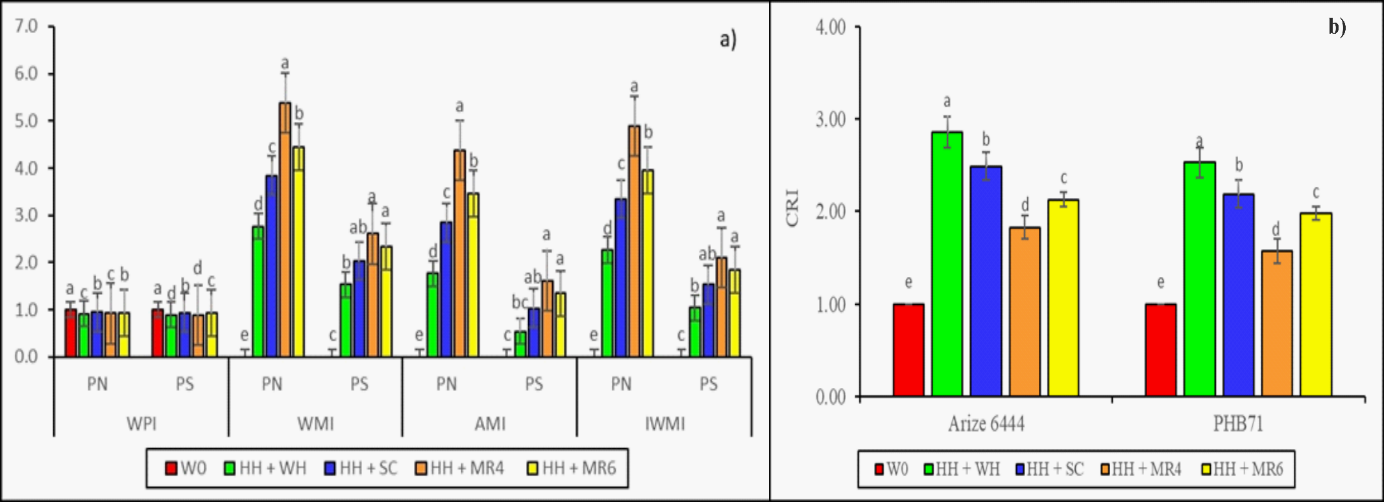
**

**Figure S2.** Interaction effect of planting geometry × weed management on WPI, WMI, AMI, IWMI (a) and cultivar × weed management on CRI (b) in rice. P_N_, sowing with seed drill at 18.5 cm row spacing; P_S_, square planting at 25 cm× 25 cm row to row and plant to plant spacing; W_C_, weedy check (no weed management); H_H_+W_H_, single hand hoeing at 12 DAS *fb* one hand weeding at 30 DAS; H_H_+S_C_, single hand hoeing at 12 DAS *fb* *Sesbania aculeata* co-culture and mulched 45 DAS; H_H_+M_R4_, one hand hoeing at 12 DAS *fb* rice residue mulching @ 4 t ha^-1^; H_H_+M_R6_, single hand hoeing at 12 DAS *fb* rice residue mulching @ 6 t ha^-1^. Means with different alphabets are significant (p<0.05).

**
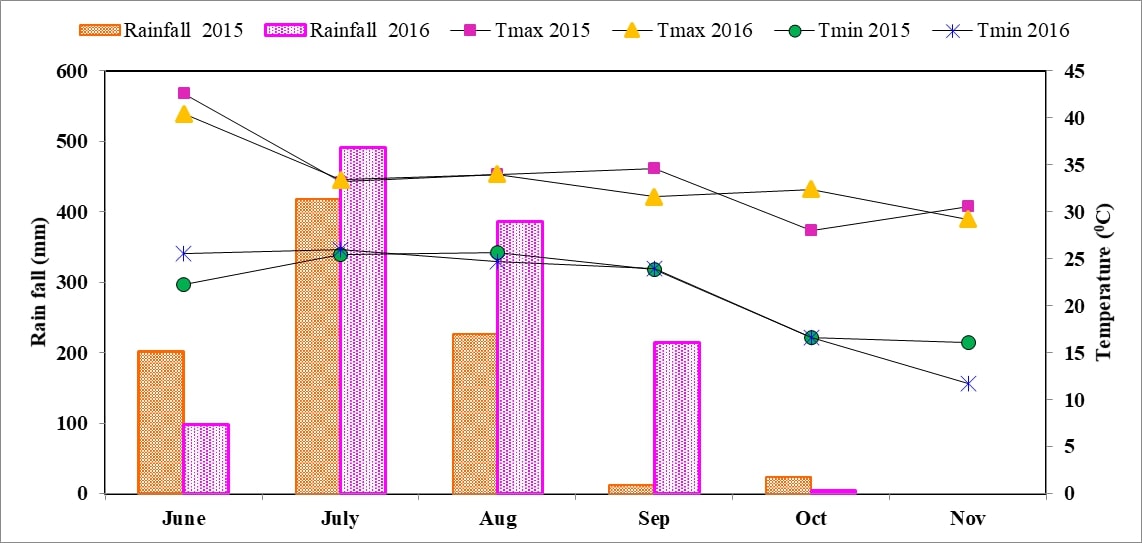
**

**Figure S3.** Monthly rainfall (total), mean maximum and mean minimum temperature during crop season.

**Table legends**

**Table S1**. Interaction effect of planting geometry and non-chemical weed management on grain yield of rice (t ha^-1^).

**Table S2.** Interaction effect of cultivar and non-chemical weed management on grain yield of rice (t ha^-1^).

**Table S3.** Cost of inputs used in this study.

| **Weed management (WM)** | **Planting geometry (PG)** | |
| --- | --- | --- |
|  | **P_N_** | **P_S_** |
| W_0_^†^ | 1.84 d* | 2.47 d |
| H_H_*+*W_H_^†^ | 4.74 a | 4.94 a |
| H_H_*+*S_C_^†^ | 4.58 a | 4.77 a |
| H_H_*+*M_R4_^†^ | 3.94 c | 4.16 c |
| H_H_*+*M_R6_^†^ | 4.34 b | 4.56 b |
| *P* value | 0.0210 | 0.0210 |

**Table S1.** Interaction effect of planting geometry and non-chemical weed management on grain yield of rice (t ha^-1^). *Means with different alphabets are significant (p<0.05). P_N_, sowing with seed drill at 18.5 cm row spacing; P_S_, square planting at 25 cm× 25 cm row to row and plant to plant spacing; W_C_, weedy check (no weed management); H_H_+W_H_, single hand hoeing at 12 DAS *fb* one hand weeding at 30 DAS; H_H_+S_C_, single hand hoeing at 12 DAS *fb* *S. aculeata* co-culture and mulched 45 DAS; H_H_+M_R4_, single hand hoeing at 12 DAS *fb* rice residue mulching @ 4 t ha^-1^; H_H_+M_R6_, single hand hoeing at 12 DAS *fb* rice residue mulching @ 6 t ha^-1^.

| **Weed management (WM)** | **Cultivar** | |
| --- | --- | --- |
|  | **Arize 6444** | **PHB71** |
| W_0_^†^ | 2.26 d* | 2.52 d |
| H_H_*+*W_H_^†^ | 5.13 a | 4.56 a |
| H_H_*+*S_C_^†^ | 4.96 a | 4.40 a |
| H_H_*+*M_R4_^†^ | 4.15 c | 3.95 c |
| H_H_*+*M_R6_^†^ | 4.71 b | 4.20 b |
| *P* value | 0.0246 | 0.0246 |

**Table S2.** Interaction effect of cultivar and non-chemical weed management on grain yield of rice (t ha^-1^). *Means with different alphabets are significant (p<0.05). P_N_, sowing with seed drill at 18.5 cm row spacing; P_S_, square planting at 25 cm× 25 cm row to row and plant to plant spacing; W_C_, weedy check (no weed management); H_H_+W_H_, single hand hoeing at 12 DAS *fb* one hand weeding at 30 DAS; H_H_+S_C_, single hand hoeing at 12 DAS *fb* *S. aculeata* co-culture and mulched 45 DAS; H_H_+M_R4_, single hand hoeing at 12 DAS *fb* rice residue mulching @ 4 t ha^-1^; H_H_+M_R6_, single hand hoeing at 12 DAS *fb* rice residue mulching @ 6 t ha^-1^.

| **Particular** | **Cost** |
| --- | --- |
| Rice seed (US$ kg^-1^) | 4.10 |
| *Sesbania* seed (US$ kg^-1^) | 0.45 |
| Urea (US$ kg^-1^) | 0.10 |
| Diammonium phosphate (US$ kg^-1^) | 0.33 |
| Murate of Potash (US$ kg^-1^) | 0.21 |
| Zinc sulphate (US$ kg^-1^) | 0.37 |
| Labour wages (US$ person^-1^day^-1^) | 4.39 |
| Irrigation (US$ hr^-1^) | 0.55 |
| Tractor cost (US$ hr^-1^) | 5.97 |
| Minimum support price of rice grain (US$ kg^-1^) | 0.23 |
| Market price of rice straw (US$ kg^-1^) | 0.04 |
| Land rent (US$ ha^-1^season^-1^) | 1.79 |
| Interest on working capital (percent annum^-1^) | 15.00 |

**Table S3.** Cost of inputs used in this study
